# Development of a Cohesive Predictive Model for Substance Use Disorder Rehabilitation Using Passive Digital Biomarkers, Psychological Assessments, and Automated Facial Emotion Recognition: Protocol for a Prospective Cohort Study

**DOI:** 10.2196/71374

**Published:** 2025-06-27

**Authors:** Andrea P Garzón-Partida, Kimberly Magaña-Plascencia, Diana Emilia Martínez-Fernández, Joaquín García-Estrada, Sonia Luquin, David Fernández-Quezada

**Affiliations:** 1 Departamento de Neurociencias Centro Universitario de Ciencias de la Salud Universidad de Guadalajara Guadalajara Mexico; 2 Instituto de Neurociencias Traslacionales Centro Universitario de Ciencias de la Salud Universidad de Guadalajara Guadalajara Mexico; 3 Departamento de Farmacobiologia Centro Universitario de Ciencias Exactas e Ingenierías Universidad de Guadalajara Guadalajara Mexico

**Keywords:** digital biomarker, predictive model, substance use disorder, machine learning, facial emotion recognition, digital phenotype, digital health, wearable, data model, real-time monitoring

## Abstract

**Background:**

Substance use disorder (SUD) involves excessive substance consumption and persistent reward-seeking behaviors, leading to serious physical, cognitive, and social consequences. This disorder is a global health crisis tied to increased mortality, unemployment, and reduced quality of life. Altered brain connectivity, circadian rhythms, and dopaminergic pathways contribute to sleep disorders, anxiety, and stress, which worsen SUD severity and relapse. Factors like trauma and socioeconomic disadvantages heighten risk. Digital health technologies, including wearables and machine learning, show promise for diagnosis, monitoring, and intervention, from relapse prediction to early detection of comorbidities. With high relapse rates and younger patient cases, these innovations could enhance the treatment outcomes of SUD.

**Objective:**

The objective of this study is to develop and validate a predictive model with machine learning for the duration of therapy and the rehabilitation or relapse in patients with SUD, using digital physiological measurements, psychological profiles, automatic facial emotion recognition, and the emotional state during craving.

**Methods:**

The study will be conducted with adult male patients with SUD at a rehabilitation center and control volunteers. Participants will undergo a self-reported demographic and psychological assessment, a clinician-administered craving and emotional reaction test, and will also be monitored using a smartwatch. SUD participants will be monitored for a total of 18 months (6 months during rehabilitation, an additional 12 months post discharge), and control participants for a total of 6 months. All participants will be reassessed at the sixth month of monitoring. The collected data will then be used to train models with a neural network, which will then be validated against other models and compared with other algorithms. Demographic, psychological, digital biomarkers, and craving profiles will be created, correlations will be analyzed, and they will be compared with controls to generate a digital phenotype of SUD. When the model achieves adequate validity (area under the curve of ≥0.80), a graphic user interface will then be designed for clinical use.

**Results:**

The study is supported by the Program for the Improvement of Working Conditions for Members of the SNII and SNCA (PROSNII U006EST), and APPAC-VII-CUCS-2025 for Article Publication Fees, from the University of Guadalajara. The research protocol was approved by the University of Guadalajara (reference CI-01225) in January 2025. Recruitment of patients with SUD and control participants will take place from January 2025 through January 2027.

**Conclusions:**

As shown in recent studies, accessible and affordable wearables, like commercial smartwatches, combined with psychological, demographic, and emotional state data, used with a machine learning predictive model, may be able to be used as tools to enhance SUD rehabilitation and prevent relapse.

**International Registered Report Identifier (IRRID):**

PRR1-10.2196/71374

## Introduction

Substance user disorder (SUD) is characterized by the excessive consumption of a substance, persevering and repetitive conducts of reward-seeking and reinforcing, regardless of the harmful physical, cognitive, behavioral, and social consequences. The disturbances created by SUD are related to alterations in brain connectivity, even after successful remission of substance use [1].

Worldwide, SUD is considered a multidimensional and critical health crisis phenomenon, connected with an increase in mortality, development of diseases, reduction of safety, increase in unemployment rates, and reduction of quality of life [2].

Research has suggested a link between the alteration of circadian rhythms and SUD in its earlier stages, that in time, due to the alteration of dopaminergic and glutamatergic pathways [3], results in sleep disorders. The development of these has been found to be a pivotal factor in the progression, severity, and relapse of SUD. These findings confirm a bidirectional relationship between SUD and sleep disorders [4-6].

Increased levels of anxiety and stress have also been associated with SUD, specifically in the abstinence and craving stages of the addiction cycle [7-12]. Anxiety and stress have also been related to an increase in heart rate, sweating, oxygenation levels, and atypical patterns of physical activity, all of which have been found in SUD [13-17].

In addition to physiological biomarkers, psychological evaluation has identified critical changes in executive function, decreased emotional regulation, and heightened levels of anxiety and depression [18-22]. Furthermore, environmental factors have been recognized to increase the risk of developing this disorder, like adverse experiences during childhood, violent or traumatic events, and disadvantageous economic conditions [23-29].

The first studies using digital technologies in mental health disorders were done on patients with major depressive disorder (MDD) through ecological moment assessment questionnaires through mobile devices, increasing a better treatment adherence [30-33]. After this study, various researches have been carried out, using varying methodologies and with objectives going from early diagnosis to prognosis of various complex pathologies, such as Alzheimer disease and major neurocognitive disorders some of the areas, where wearables and virtual reality have been used, allowing to observe cognitive decline and behavioral patterns [34-39].

Machine learning (ML) has also been used as a tool for the diagnosis of anxiety disorders with the use of speaking patterns, gaming activities, and self-reports [40-49]. It has also allowed researchers to measure the severity and variability of that disorder in varying environments through wearables, allowing remote intervention and through simulations [30,48,50-52]. It has also been used in predictive models for early depression diagnosis through smartphone use, severity, and progression through physical activity, sleep, and social interaction data [53-61]. In addition, ML models (MLM) have been trained to detect relapse in MDD and risk of suicide [62-64]. Similarly, it has been used to predict the start of a psychotic episode 5 days before it by using patterns of movement and sleep disturbance [63,65].

With a 60% relapse rate, an average of 5 recovery attempts required for full remission, and a growing number of new patients with SUD presenting at younger ages [2], this disorder represents a significant opportunity for digital health technologies. These tools, often used in managing cognitive and mood disorders commonly comorbid with SUD, have the potential to enhance treatment effectiveness and reduce relapse rates.

This study aims to integrate physiological, behavioral, and psychological data through digital technologies to develop comprehensive digital profiles of individuals with SUD. These profiles will be analyzed using ML tools to identify clinically relevant patterns. The resulting models are intended to serve as complementary tools in clinical practice, supporting treatment decisions, improving prognostic accuracy, and facilitating the monitoring of patients in remission.

## Methods

### Goal and Objectives

The purpose of this study is to develop and validate a predictive model of substance use relapse or rehabilitation with ML in patients with SUD. This will be through using digital physiological measurements (digital biomarkers), demographic and psychological profiles, automatic facial emotion recognition, and the emotional state during craving (ESDC).

### Study Design

This study adopts a prospective cohort design, using data collected from nontraditional experimental groups to develop a MLM aimed at predicting clinical outcomes in the rehabilitation or relapse of patients with substance use disorders. The model will rely on comparative analyses between groups, identifying patterns in physiological, psychological, and emotional reactivity data to enhance predictive accuracy.

No interventions will be implemented in either group as part of this study, ensuring that all collected data reflect naturalistic conditions and minimizes external influence on the observed outcomes.

### Participant Identification and Randomization

Participants in both groups will be randomly assigned a unique identification number, which will be used throughout the study to ensure anonymity and meet the inclusion criterion of randomization. Psychological assessments conducted at the rehabilitation center will be conducted through self-reported questionnaires, administered in a traditional pen-and-paper format, overseen by the institution’s psychologists, who will use only the assigned ID numbers during the digitization of all data. Control participants will complete all psychological tests in a self-reported computer-based format, using their ID numbers, which will be randomly assigned at the start of the monitoring period.

A secondary randomization process will be conducted to create k-clusters of data before training the ML algorithm. This step will also include the selection of test data for verification purposes, ensuring robust validation of the model.

### Blinding

Due to the nature of this study, neither the participants nor the first group of research staff will be blinded to group assignments. However, the group allocation of participant ID clusters will remain concealed from the second group of researchers. This second group will be responsible for data management, training the ML algorithm with the predictive model, and conducting validation, verification, and testing of the highest-performing models.

### Timeline

This study will include 2 types of observations: continuous passive monitoring and active surveys conducted at specific time points. Passive monitoring will occur continuously for 6 months in both groups, followed by an additional year for participants with SUD after their discharge from the treatment facility. Active surveys will be conducted at three time points: (1) baseline session or baseline, (2) 3 months after baseline, and (3) 6 months after baseline.

**Figure 1:**
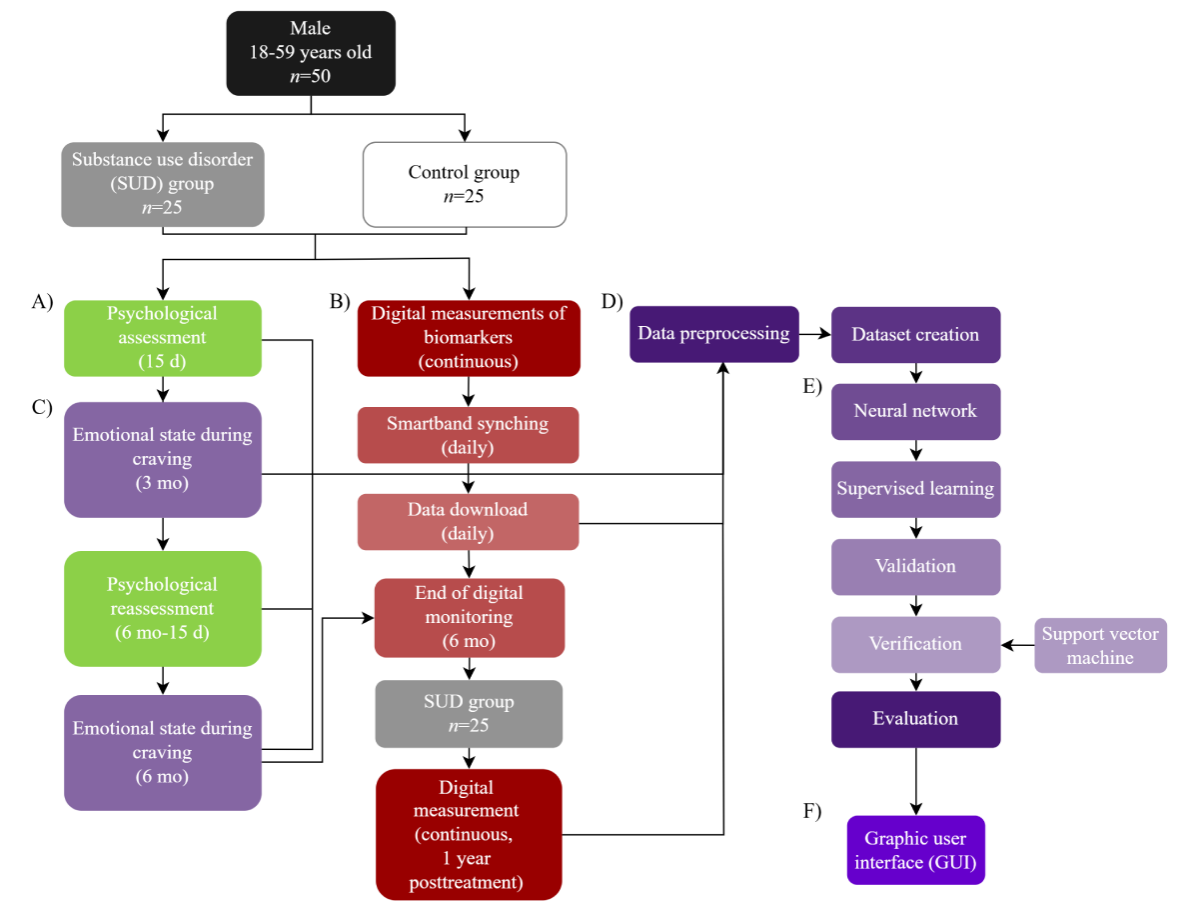
Timeline of experimental procedures. (A) Demographic and psychological assessment, a battery of interviews and tests will be applied at 2 timepoints. (B) Digital measurements with the smart band Xiaomi Redmi Smart Band 8, starting on day one for controls, and after the first month of treatment for substance use disorder. (C) Evaluation of the emotional state during craving halfway through the digital monitoring period (third month), and at the end of it. (D) Data preprocessing is required for all 3 data sources. (E) Training in a machine learning predictive model in neural networks. (F) creation of a graphic user interface for clinical use.

For digital monitoring purposes, smart bands provided to participants will be synchronized with a smartphone at the baseline session. Psychological assessment will be conducted within the first 15 days after baseline. Retesting will take place during the 15 days prior to the 6 months after baseline. Emotional reactions to cravings will be assessed at two points: (1) 3 months after baseline and (2) at 6 months after baseline (Figures 1A-1F).

### Participants and Study Setting

#### Sample Size

The minimum sample size was determined using Epidat 4.2 software (Xunta de Galicia), following the equation for estimating the comparison between independent means. The calculation was based on data reported by Rykov et al [30], in a study where patients were screened using wearable devices, and (MLMs were applied (Textbox 1).

**Sample characteristics.**

- Variance: distinct
- Standardized mean difference=2.54
- Expected SD of population 1=1.6
- Expected SD of population 2=3.21
- Ratio between sample sizes=1
- CI=95%
- Statistical power=80%

In addition, based on the rule of one-to-ten for artificial neural network (ANN) models suggested by Kavzoglu and Mather [31], in which a sample should be at least 10-100 times the numbers of independent variables (4 in this study, based on the 4 suggested profiles), a sample of 40-400 participants was obtained. Therefore, a minimum sample size of 25 was determined for each group. Participants will be recruited within 2 years of the beginning of the study, aiming for recruitment greater than 25 per group.

#### Recruitment

Participants for the SUD group will be recruited from a male-only, age-restricted inpatient drug rehabilitation facility in Cajititlán, Mexico. Eligibility criteria include being male, aged 18-59 years, and actively participating in a therapeutic community treatment program for SUD, with a cognitive behavioral therapy approach. Furthermore, candidates must undergo their first rehabilitation attempt within this specific program and be under medication management for any possible mental health comorbidity diagnosed with the Mini International Neuropsychiatric Interview (MINI) or physical disease. Finally, participants are required to review the privacy and safety terms and provide informed consent before joining the study. Smartwatches will be synchronized with a community smartphone by the personnel of the rehabilitation center, in accordance with the restricted access to smartphones.

#### Control Participants

Control group participants will be recruited through an open call for volunteers in the ZMG area, restricting the sampling to similar sex and age characteristics of the patients from the rehabilitation facility. To be eligible, individuals must be male, aged 18-59 years, have no history of SUD, either diagnosed or self-reported, with no current use of substances, excluding caffeine. They must review and agree to the privacy and safety terms, as well as the requirements for active participation. In addition, participants are required to commit to a six-month study period, synchronize and upload their data weekly, and attend scheduled meetings with the research team. In case an individual is interested in participating in the study, but does not have access to a personal smartphone, a device will be provided.

#### Exclusion Criteria

The exclusion criteria have been established for both groups equally. Individuals with physical conditions that significantly affect mobility, impede the use of a smart band or the registry of facial expressions, such as facial (eg, craniofacial abnormalities, facial nerve palsies, or severe neurological conditions) or neurological disorders, are ineligible. In addition, patients with a history of heart conditions, breathing issues, as well as significant vision and hearing impairment will be excluded, as these conditions might affect the measurements.

#### Withdrawal Criteria

Participants will be withdrawn from the study under the following circumstances: failure to use or misuse of the smart band for more than 5 consecutive days or exceeding 20% of the six-month period will result in the cessation of monitoring, with participants being notified, the device retrieved, and the data discarded. Relapse in substance use for the SUD group or the onset of addictive behavior for controls will also lead to immediate withdrawal; if the exact timing of such behavior cannot be determined, the corresponding data will be excluded. Finally, participants who voluntarily drop out of the study or request the removal of their data will be withdrawn, and their data will be discarded.

### Study Setting

Participants will be evaluated in person at the key time points outlined in Figure 1 by health care professionals trained in conducting clinical interviews, administering psychological tests, and assessing the ESDC. For patients with SUD, assessments will take place in a designated area within the rehabilitation treatment facility, ensuring both privacy and comfort. Control group participants will be assessed at the University Center of Health Sciences, University of Guadalajara, in Guadalajara, Mexico, by trained health professionals.

Passive monitoring of biological and digital measurements using a smart band will be conducted for at least 22 hours per day, allowing a threshold of 2.4 hours (20%) of lost daily data, 7 days a week. Participants will be required to wear the device during sleep. Participants’ physiological activity will be recorded during various activities and across diverse locations.

### Data Collection, Storage, and Protection

Digital measurements will be downloaded from a Xiaomi account as a password-protected zip file. The csv files containing data from the linked devices will be processed and coded before being securely stored for future use. Clinical data will be entered into the database under a unique study ID number, either directly from source materials or from the instruments used for assessment.

In compliance with ISO (International Organization for Standardization)/IEC (International Electrotechnical Commission) 29100 guidelines and the General Data Protection Regulation (GDPR) [66,67], all data will be stored in encrypted storage units housed within the Department of Neuroscience at CUCS, University of Guadalajara. In addition, all files will be backed up on an encrypted cloud system hosted by the University of Guadalajara servers. During the study, access to these databases will be restricted to authorized study staff with the necessary training and permissions, ensuring the privacy and confidentiality of participants.

### Instruments

The study instruments that will be used in this protocol to measure each assessment category referred to in the timeline (Figure 1), are presented in Table 1.

**Table 1:** Study instruments.

Instrument	Period				
	Baseline	3 Months	6 Months	18 Months	Continuous
Wearable: Xiaomi Redmi Smart Band 8^a^	✓	✓	✓	✓ ^b^	✓
Demographic survey^c^	✓				
The Adverse Childhood Experiences Assessment (ACEs)^d^	✓				
The University of Rhode Island Change Assessment Scale (URICA)^d^	✓				
The Alcohol, Smoking and Substance Involvement Screening Test (ASSIST)^d^	✓				
The Beck Depression Inventory (BDI-II)^d^	✓				
The Beck Anxiety Inventory (BAI-II)^d^	✓				
The Pittsburgh Sleep Quality Index (PSQI)^d^	✓		✓	✓^b^	
Wechsler Adult Intelligence Scale IV (WAIS-IV): Digit Span and Letter-Number Sequencing subscales^d^	✓		✓		
Emotional Pictures Database (EmoMadrid)^e^		✓	✓		
Mannheimer Craving Scale (MaCS)^e^	✓	✓	✓		
Automatic facial emotion recognition software (FER)^e^		✓	✓		

^a^Digital measurement of biomarkers.

^b^Substance use disorder group only.

^c^Demographic assessment.

^d^Psychological assessments.

^e^Emotional state during craving assessment.

### Digital Measurement of Biomarkers

The Xiaomi Redmi Smart Band 8 will be worn on the user's nondominant hand, positioned 2 cm below the wrist bone and secured at a comfortable level, as instructed by the user manual. The device will be worn for a minimum of 22 hours per day, allowing for an anticipated loss of up to 2 hours of data due to activities such as recharging the device, synchronization with a smartphone, showering, and other routine tasks.

Physiological data, including heart rate (HR), peripheral capillary oxygen saturation, physical activity, stress, and sleep, will be collected using the device's shortest available measurement intervals. Data will capture periods of both high and low activity, during self-reported exercise, regular physical activity, and rest, and will identify any abnormalities in activity patterns.

**Figure 2:**
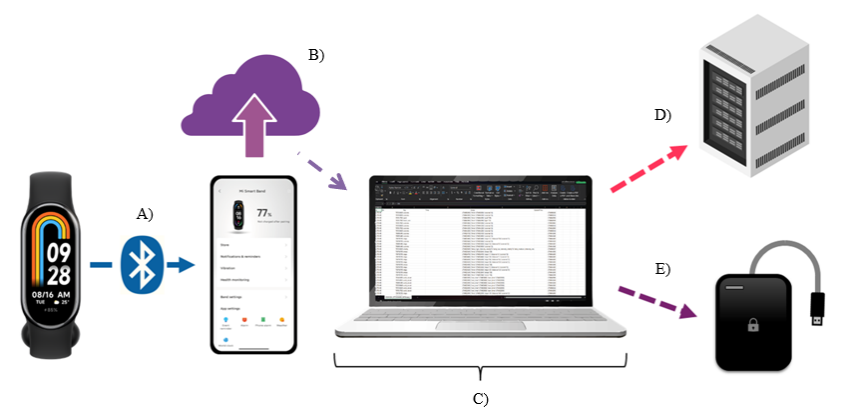
Scheme of smart band data retrieval. (A) Data from the smart band Xiaomi Redmi Smart Band 8 via Bluetooth to the smartwatch it’s been linked to for synchronization. (B) Through the Xiaomi app Mi Fitness, the data of the monitored period will be uploaded to the account’s cloud to be downloaded by the researchers. (C) Raw data will be extracted, labeled, and preprocessed. (D) All data will be saved in an assigned encrypted server, property of Universidad de Guadalajara. (E) Alternatively, a second copy will be done on an encrypted external hard drive.

Data synchronization, which facilitates upload to the associated Xiaomi account, will occur every 72 hours for all participants during the 6- or 18-month study period, as applicable. This data will be downloaded, preprocessed, and stored securely on an encrypted external hard drive and the institution's server for access by authorized research staff within 48 hours (Figures 2A-2E). Participants will receive a reminder notification if synchronization is not completed within 5 days.

Raw data will be used to compute digital the physiological features proposed in a MLM study by Rykov et al, [30] (Table 2).

**Table 2:** Summary of raw measurements and physiological features.

	Raw data	Physiological features
Physical activity	Calories, steps, vitality, and time standing up	Daily footsteps, sedentary time, light physical activity, moderate physical activity, and vigorous physical activity
Heart rate (HR)	Beats per minute (BPM), maximum and minimum BPM, and single measure	HR resting HR, daytime HR, nighttime HR, HR delta, and HR root-mean-square successive differences
Sleep	Type of sleep, wake-up time, bedtime, and awake	Coefficient variance, time in bed, total sleeping time, sleep efficiency, sleep onset latency, waking after sleep onset, and sleep midpoint
Rhythm	Activity peaks	Circadian rhythm: COSINOR and nonparametric measurements
Oxygenation	SpO_2_^a^ during daytime, single measures of SpO_2_, and sleep time SpO_2_	—^b^
Stress	Stress level and single stress measurements	—

^a^SpO₂: peripheral capillary oxygen saturation.

^b^Not available.

### Demographic and Psychological Assessment

Demographic information, such as age, place of birth, education, occupation, civil status, household income, housing characteristics, as well as medical and family history, will be collected at baseline. A form will be filled in by the participant, assistance from the trained research staff will be provided if it is required.

The psychological tests will be administered at baseline within the first 15 days of participation. A battery of self-report questionnaires has been chosen to assess for a history of childhood trauma (Adverse Childhood Experiences Assessment [ACEs]) [68], the presence of depression or depressive symptoms (Beck Depression Inventory [BDI-II]) [69], anxiety and stress (Beck Anxiety Inventory [BAI-II]) [70], sleep disorders (Pittsburgh Sleep Quality Index [PSQI]) [71], and short-term memory (Wechsler Adult Intelligence Scale IV [WAIS-IV] subtests: Digit Span and Letter-Number Sequencing) [72]. Substance use–specific tests will aim to determine the type of substance, frequency and quantity of used drugs (Alcohol, Smoking and Substance Involvement Screening Test [ASSIST]) [73], level of substance craving (Mannheimer Craving Scale [MaCS]) [74], as well as the stage or frame of mind of the individual starting the therapeutic process (University of Rhode Island Change Assessment Scale [URICA]) [75].

**Figure 3:**
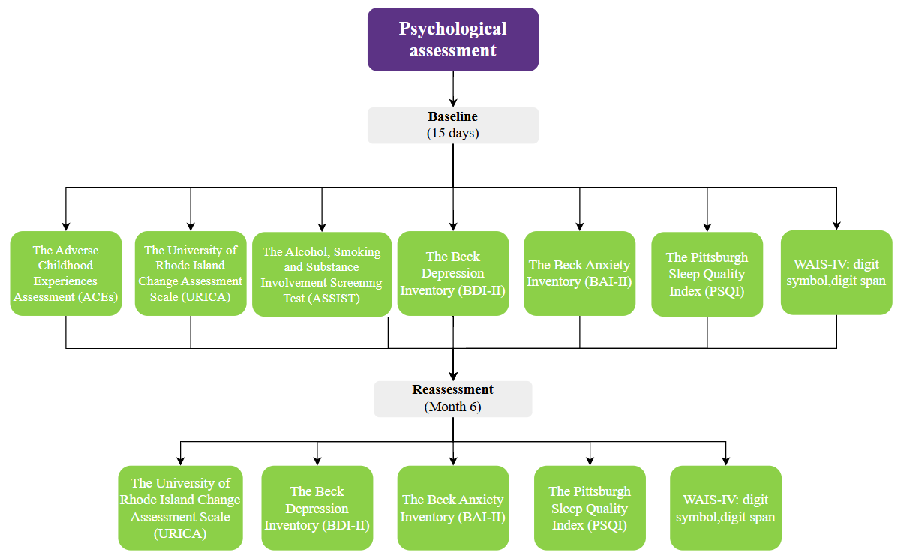
Flowchart of the psychological test battery. WAIS-IV: Wechsler Adult Intelligence Scale IV;.

To obtain a comparative measurement after treatment, depression, anxiety and stress, sleep disorders, short-term memory, stage of change and craving, will be reevaluated within fifteen days before the sixth month period of digital tracking ends (Figure 3).

### Emotional State During Craving

Given the significance of craving in SUD, a profile of the ESDC will be developed. Participants will be shown images from the Emotional Picture Database (EmoMadrid), designed by CEACO, which will be used as stimuli to elicit an emotional response [76]. Participants will be recorded all throughout the test, and Facial Emotion Recognition (FER) software. Software will provide an objective, real-time measure of participants’ emotional expressions, which we will analyze in conjunction with their self-reported “Emotional State during Craving” data. The FER software is a personal script written in python, which automatically detects emotions by analyzing changes of facial expression of dataset that consists of 48×48 pixel grayscale images (32,298 images) of faces (FER-2013). Craving levels will be measured both before and after the test.

An experimental setup will be arranged to ensure optimal conditions for the test. This setup will include a white screen for image projection, an HD camera for recording the participant, and a controlled environment with low lighting and sound insulation to minimize distractions. A trained researcher will administer the test in this environment.

At the start of the experiment the FER software will be activated and participants will respond to items 9 to 11 of the MaCs scale (Table 1), which assesses craving-related anxiety levels, the effort required to resist urges, and impulses to consume substances. Following this brief questionnaire, the instructions for the experiment will be presented both visually and verbally to ensure the participant's full understanding.

A modified version of the protocol has been designed to suit the characteristics of the study population. Fifty images have been selected and categorized as positive, negative, neutral, or substance-use-related. These images have been further customized to align with the substance use profile of the participant group. Each image will be displayed for 1 second, after which participants will score it based on valence and activation. Once a score is provided, the next image will be shown. The procedure will be divided into 2 sets of 25 images, with a 30-second rest period between sets.

**Figure 4:**
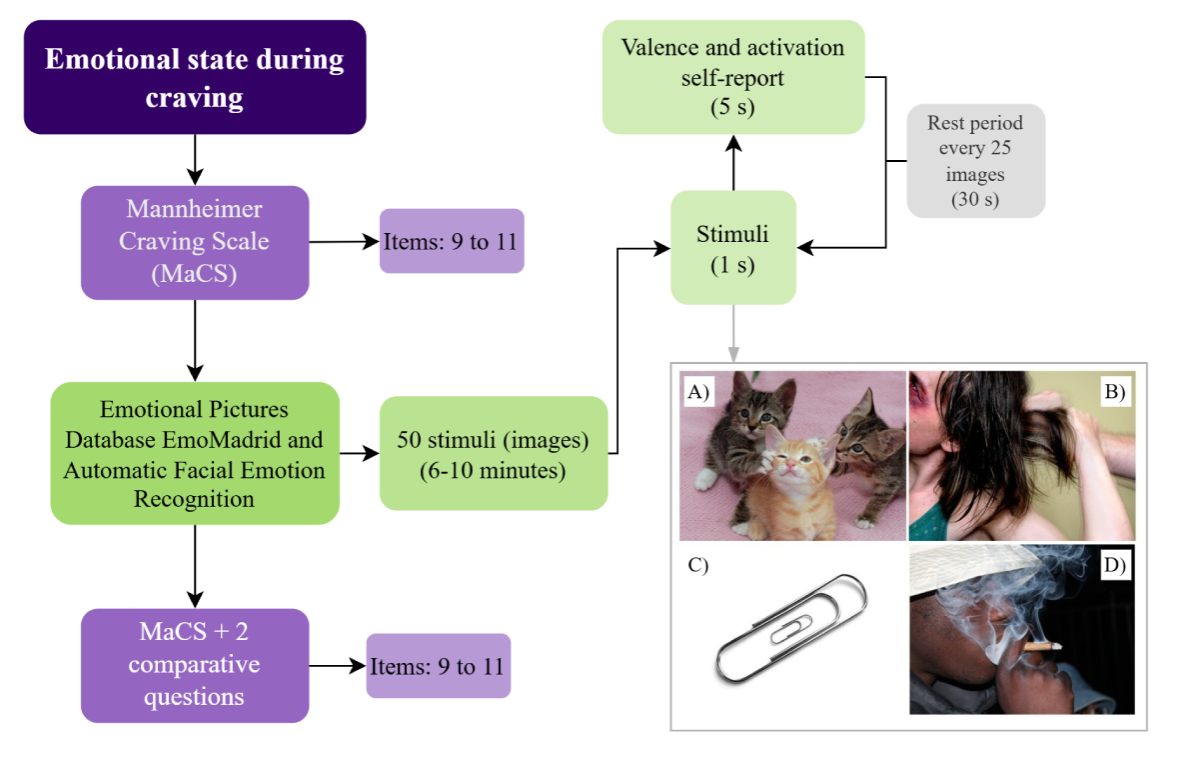
Flowchart of emotional reaction to craving assessment. Steps of the brief craving assessment, in addition to a modified version of the emotional pictures database for valence and activation, including a sample of the chosen images of each category. (A) Positive images (n=15). (B) Negative images (n=16). (C) Neutral images (n=10). (D) Substance use-related images (n=9).

After the image presentation, participants will be reassessed using the same 3 MaCs scale items. In addition, they will be asked whether they experienced any changes in their emotional state after viewing the images and to provide a qualitative description of any observed shift (Figures 4A-4D).

All data obtained from the FER analysis, MaCS EMA, the EmoMadrid emotional response assessment and physiological data obtained by the smart band, will be used to observe the relationship between exposure to emotional and substance use visual stimuli, emotional response and craving.

### Statistical Analysis

Statistical analysis will be conducted using R-Studio IDE (version 4.3.1; Posit PBC), Prism GraphPad (version 9, GraphPad Software Inc), and MATLAB (version R2025a, MathWorks Inc). A descriptive analysis of normality (Shapiro-Wilkin normality test) means and medians, SD and error, variance (ANOVA or Kruskal-Wallis tests) will be performed. Correlation analyses (Spearman or Pearson) will be conducted to gain a preliminary understanding of the interactions between variables, as well as using time series analysis descriptive models to identify patterns, trends and cycles of activities by participants, and by groups. MATLAB will be used for advanced data processing and visualization, ensuring robust and comprehensive analysis.

### Data Exclusion

Missing data will be addressed through imputation. If more than 20% of daily data samples are missing, they will be excluded. For missing data below this threshold, linear interpolation and neighbor mean imputation (k-nearest neighbor) will be applied during preprocessing using MATLAB.

The quality of data collected from the smart band will be assessed by correlating heart rate at randomly selected timestamps with oxygen saturation, activity levels, self-reported exercise, and sleep type during sleep hours. If atypical correlations are identified, 10 data points before and after the anomaly, along with 5 additional randomly chosen timestamps, will be reviewed. A day with more than 20% of faulty measurements will be excluded from the dataset.

Data will also be excluded in cases of device misuse, such as exchanging the device with other participants or nonparticipants, switching the wearing side without prior notice, or removing the device for more than 5 hours without informing the research team.

If the total sum of missing and excluded data exceeds 20% of the 6-month period, the participant's data will be removed from the study. Finally, for SUD participants, any measurements obtained during periods of substance use will be excluded, and their participation in the study will be terminated.

### MLMs

Predictive MLMs for classification and regression will be designed, trained, and tested following the methodology outlined by Luo and colleagues [77] in their Guidelines for Developing and Reporting Machine Learning Predictive Models in Biomedical Research.

### Data Preprocessing

Databases and labeling will initially be managed using Microsoft Excel (version 2412), after which MATLAB (version 2025a, MathWorks, Inc) will be used for data preprocessing. This process will include data cleaning and preparation to ensure the dataset is suitable for ML training.

Feature selection will be done following a filter methodology after correlation analysis to assess the correlation with the target and statistical measures. All data obtained through the wearable device, psychological assessment and ESDC will be scaled through a *z*-score standardization for dimensionality reduction and integration across all modality.

The data will be divided into training, validation, and test datasets using a k-fold cross-validation approach. The dataset will be split into k folds, with 2 folds randomly selected: one for validation and another for testing. The remaining k-2 folds will be used for training. The number of folds will vary depending on the specific model, enabling a broader experimental variance and improved model evaluation.

### Algorithms

Modeling will be conducted using MATLAB, with ANN serving as the base algorithm. Additional algorithms, including decision trees, random forests, and support vector machines, will be used for validation. This multialgorithm approach will facilitate a comprehensive comparison of predictive accuracy across various models.

### Output

**Figure 5:**
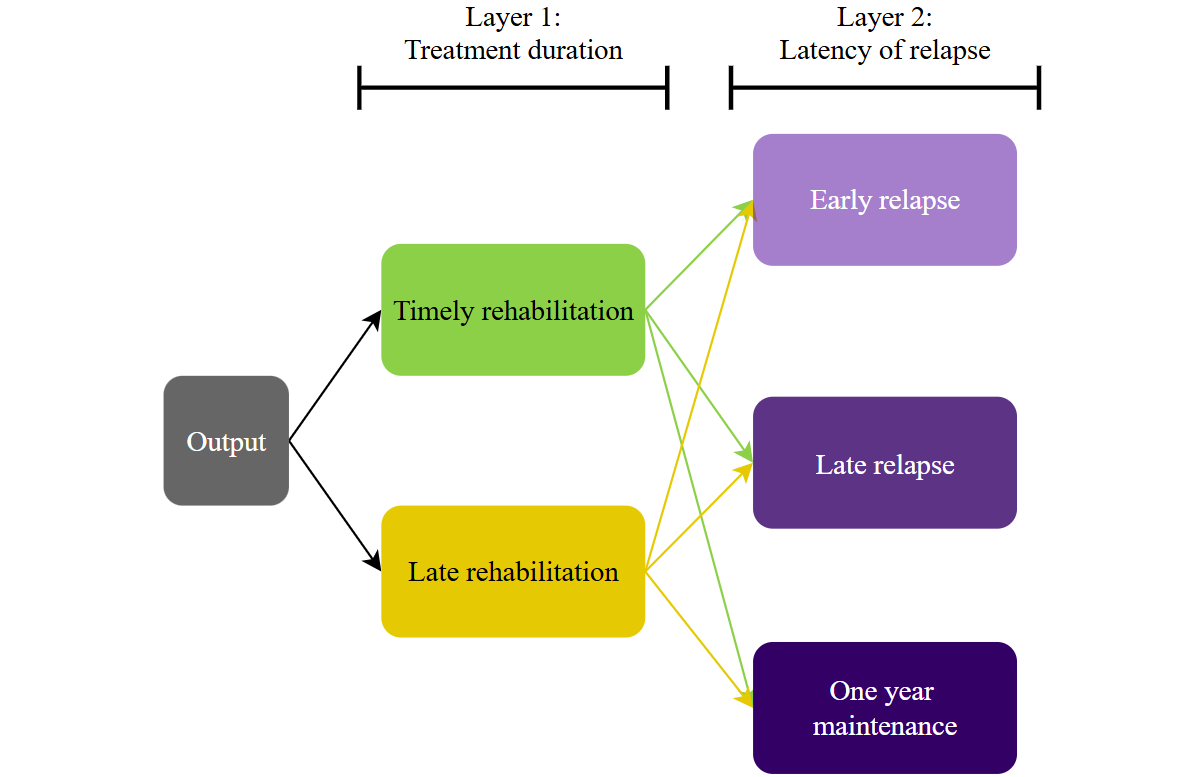
Model’s possible outputs. These categories will be used as output labels of the predictive model and will allow the evaluation of the patterns detected from the input.

The primary objective of this study is to develop a MLM capable of predicting clinical outcomes in patients. To achieve this, the data will be segmented and classified into 5 categories (Figure 5). Around 2 of these categories will correspond to the first layer, which pertains to the duration of the therapeutic process, while the remaining 3 will focus on the latency of relapse, if any.

For the duration of the therapeutic process, the following labels will be used:

1. Timely rehabilitation: patients with SUD who complete the rehabilitation protocol within 7 months.
2. Late rehabilitation: patients with SUD who complete the protocol in 7 months or longer.

Regarding relapse latency, the labels will include the following:

1. Early relapse: substance use occurring within the first 6 months post discharge.
2. Late relapse: substance use occurring after 6 months but before the completion of 18 months post discharge.
3. One-year maintenance: patients who have abstained from substance use at the end of the 18-month postrecovery period.

### Validation and Verification

All models and algorithms will undergo validation, verification, and testing using MATLAB’s tools for model evaluation and hyperparameter optimization. These processes will assess precision, performance, and recall metrics. Optimal model performance will be defined by achieving an area under the curve (AUC) of ≥0.80 and F2 score of ≥0.8.

The output categories will serve as benchmarks to evaluate the validity, reliability, and precision of the models. The validation dataset will be used to perform initial comparisons both within individual models of each algorithm and across different algorithms, replicating models with distinct architectures to refine performance.

For model verification, the test dataset will be used to determine final AUC and F2 scores. This final evaluation will confirm the models’ effectiveness as ML tools for predicting rehabilitation outcomes and informing clinical decision-making.

### Outcomes

#### Profiles

A total of 4 distinct subprofiles will be generated based on the results of the statistical analysis: (1) demographic, (2) psychological, (3) craving, and (4) digital biomarkers. These profiles will subsequently be integrated to create digital phenotypes for individuals with SUD.

#### Predictive Model

A predictive model will be developed to distinguish between healthy individuals and patients with SUD. In addition, the model will predict the duration of a rehabilitation therapeutic program and the length of sobriety adherence for individuals rehabilitated using the center’s methodology. A minimum performance threshold of AUC≥0.8 and F2≥0.8 has been established to evaluate the model as fully functional.

#### Graphical User Interface

As an outcome of this study, a graphical user interface will be developed using MATLAB to enable practical application of the predictive tool within the rehabilitation center where this research is being conducted. The graphical user interface will facilitate clinical use of the model and allow for further training and refinement, expanding its potential utility in similar settings.

### Ethical Considerations

This study has been approved by the Research, Ethics in Research and Biosafety committees of the Centro Universitario de Ciencias de la Salud, Universidad de Guadalajara (registry number 25-06; approval ID CI01225). This certifies that the research protocol adheres to the principles outlined in the Declaration of Helsinki of ethical research involving human participants, with a satisfactory quality and relevancy of the research.

Candidates will be provided with detailed materials in both written and digital formats for review before confirming their participation. All participants will be provided with an informed consent reviewed by the previously mentioned committees, signed by the participant, researchers, and two witnesses. Participants have the right to withdraw their consent at any time and may request the removal of their data from the study, in compliance with the Federal Law on Personal Data Protection and Article 16 of the Federal Constitution of the United States of Mexico [78,79].

All identifying participant information will be anonymized through a numeric ID system. Subsequently, the obtained data will be securely stored, either locked in a filing cabinet or in encrypted digital files and retained for a minimum of 5 years before being destroyed, as required by the Official Mexican Norm for Clinical Files (NOM-004-SSA3-2012) [80]. This information will only be accessible for purposes related to contact by the research group.

Participation in this study is entirely voluntary, and no financial incentives will be provided for joining or continuing in the study.

## Results

The study is supported by the Program for the Improvement of Working Conditions for Members of the SNII and SNCA (PROSNII U006EST), and APPAC-VII-CUCS-2025 for Article Publication Fees, from the University of Guadalajara. The research protocol was approved by the University of Guadalajara (reference CI-01225) in January 2025. Recruitment of patients with SUD and control participants will take place from January 2025 through January 2027. As of June 2025, we have enrolled 35 participants, 18 patients with SUD and 17 controls. Data obtained from the device, as well as psychological and demographic data at baseline assessments and at 3 months are at the labeling and preprocessing stage of the data analysis. Results are expected to be published by the summer of 2028.

## Discussion

### Principal Findings

This study aims to evaluate the viability and efficacy of a predictive model designed to estimate the duration of a rehabilitation process and predict the likelihood of early, late, or no relapse in substance use, based on the digital phenotype of SUD. The hypothesis posits that an AUC of ≥0.80 and F2 of ≥0.8 will demonstrate that data collected from an affordable commercial smart band, demographic questionnaires, psychological tests included in the institution’s base protocol, and the proposed use of FER combined with valence and emotional activation scoring during craving episodes can effectively support the creation of this predictive model.

The potential of this project is supported by several premises. Wearable devices have become increasingly accessible and seamlessly integrated into daily life. As of January 2024, GSMA Intelligence reported 125.4 million cellular connections in Mexico, and GWI estimated that 31% of internet users aged 16-56 years use a smartwatch or smart band [81]. Considering that the device used in this study is among the most affordable commercial smart bands and compatible with any mobile operating system, the development of a viable predictive model could introduce this tool as a complementary aid in SUD rehabilitation, enhancing the effectiveness of treatment strategies.

Wearable devices and MLM have been successfully applied to various mental health conditions. For example, passive physiological activity measurements and MLMs have distinguished depressive patients from healthy controls by correlating variations in circadian rhythms, nocturnal HR, and higher depression test scores [30]. Similarly, MLMs using passive data have predicted depressive, manic, or hypomanic episodes in patients with bipolar disorder (BD) by identifying sleep disturbances, circadian rhythm changes, and physical activity variations [82]. Furthermore, facial emotion recognition combined with MLMs has been used to diagnose and predict alexithymia with an AUC of 0.80 [83]. In the context of SUD, MLMs have demonstrated potential by evaluating participant engagement, correlating stress and craving during recovery, and providing insights into treatment outcomes [84].

Psychological assessments remain a cornerstone for diagnosing and quantifying symptom severity in SUD, projecting recovery trajectories, and supporting research protocols. The psychological tests included in this study have been previously validated in SUD research, translated into Spanish, and recommended for clinical and diagnostic use [85-90]. Despite potential biases in self-reports, these tools have also been engaged in mental disorders such as schizophrenia and mood disorders like major depression and BD to develop MLMs and support symptom monitoring through digital technologies [30,46,60,91-96].

Digital tools for long-term symptom monitoring and clinical support during remission have been widely accepted in studies involving high-risk populations. For example, US veterans at risk of suicide provided valuable data on physiological activity and behavioral patterns surrounding suicide attempts through digital monitoring [64]. Similarly, high levels of acceptance have been observed among patients with BD, schizophrenia, anxiety, and major neurocognitive disorders, highlighting the feasibility of using such technologies in mental health research and clinical practice [34,37,56,60,93,97-102].

### Limitations and Considerations

This study is subject to several limitations and potential risks of bias that must be acknowledged. Participants will be selected through convenience sampling of patients with SUD in a rehabilitation facility, limiting participation to cisgender male individuals between 18 and 59 years of age in both groups, therefore limiting the generalizability of results. The experimental status will be disclosed to them beforehand as well. This awareness could influence self-reporting in psychological tests and responses during exposure to visual stimuli related to substance use, potentially introducing bias.

While the Xiaomi Redmi Smart Band 8 has been selected for its cost efficiency and suitability for future clinical applications in rehabilitation centers, its sensors may have higher measurement errors compared to research-grade devices. Studies have indicated that similar consumer-grade devices generally exhibit lower precision. However, this trade-off is justified by the need to prioritize accessibility and scalability in clinical settings.

To identify events of physical activity, such as exercise, and atypical behavior, this study will rely in self-reporting through the app included in the Xiaomi Smart Band 8 device in the control group. Activity of participants with SUD will also be assessed by classifying their behavior according to the fixed schedule at the rehabilitation facility. Data will be clustered time-wise according to the time of the day and the reported activity accordingly, to reduce possible noise created by this variability. Since these events will rely on self-reported activities, a margin of error might be present in the observations in this time window.

Data clustering by groups before final testing introduces a potential risk that research staff may infer group assignments prematurely, which could influence the interpretation of results. The reliability of data used for training the models also depends on participants’ proper use of the device and the accuracy of self-reported information, both of which carry inherent risks of bias that may impact outcomes.

The characteristics of the data and the results of ML training, validation, and verification may affect the algorithm’s performance. There is a possibility that the baseline algorithm designed for this project might not achieve the desired accuracy or generalizability, based on the participants recruited by the cutoff time of recruitment. In addition, the possibility of an algorithm better suited for smaller sample sizes could be considered at a later stage of this study based on the available data.

To address these risks, the study will follow established guidelines for developing and reporting ML predictive models in biomedical research [103]. These guidelines provide a comprehensive framework for reporting in research articles and outline structured steps for model development, aiming to reduce bias and ensure robustness in predictive modeling [77].

### Conclusion

As shown in recent studies, accessible and affordable wearables, like commercial smartwatches, combined with psychological, demographic, and emotional state data, used with a ML predictive model, may be able to be used as tools to enhance SUD rehabilitation and prevent relapse.

## Abbreviations

ACE: Adverse Childhood Experiences Assessment
ANN: artificial neural network
ASSIST: Alcohol, Smoking and Substance Involvement Screening Test
AUC: area under the curve
BAI-II: Beck Anxiety Inventory
BD: bipolar disorder
BDI-II: Beck Depression Inventory
EmoMadrid: Emotional Pictures Database
ESDC: emotional state during craving
FER: facial emotion recognition
GDPR: General Data Protection Regulation
HR: heart rate
IEC: International Electrotechnical Commission
ISO: International Organization for Standardization
MaCS: Mannheimer Craving Scale
MDD: major depressive disorder
MINI: Mini International Neuropsychiatric Interview
ML: machine learning
MLM: machine learning model
PSQI: Pittsburgh Sleep Quality Index
SUD: substance use disorder
URICA: University of Rhode Island Change Assessment Scale
WAIS-IV: Wechsler Adult Intelligence Scale IV

## References

[1] (2022). Diagnostic and Statistical Manual of Mental Disorders.

[2] World Drug Report 2023. United Nations Office on Drugs and Crime.

[3] Wise R, Robble M (2020). Dopamine and Addiction. Annu Rev Psychol.

[4] Berro LF, Roehrs T (2022). Catching up on sleep: recent evidence on the role of sleep in substance use disorders. Pharmacol Biochem Behav.

[5] Amini F, Vaziri S, Amini Z (2023). The effect of healthy mental lifestyle package on sleep quality, mental health, and lifestyle of substance users. Adv Biomed Res.

[6] Roehrs T, Sibai M, Roth T (2021). Sleep and alertness disturbance and substance use disorders: A bi-directional relation. Pharmacol Biochem Behav.

[7] Bøhle K, Otterholt E, Bjørkly SK (2023). A prospective biopsychosocial repeated measures study of stress and dropout from substance addiction treatment. Subst Abuse Rehabil.

[8] Fang Y, Sun Y, Liu Y, Liu T, Hao W, Liao Y (2022). Neurobiological mechanisms and related clinical treatment of addiction: a review. Psychoradiology.

[9] Goeders NE (2003). The impact of stress on addiction. Eur Neuropsychopharmacol.

[10] Horseman C, Meyer A (2019). Neurobiology of addiction. Clin Obstet Gynecol.

[11] Koob GF, Le Moal M (2008). Addiction and the brain antireward system. Annu Rev Psychol.

[12] Torres-Berrio A, Cuesta S, Lopez-Guzman S, Nava-Mesa MO (2018). Interaction between stress and addiction: contributions from latin-American neuroscience. Front Psychol.

[13] Hikosaka O (2010). The habenula: from stress evasion to value-based decision-making. Nat Rev Neurosci.

[14] Immanuel S, Teferra MN, Baumert M, Bidargaddi N (2023). Heart rate variability for evaluating psychological stress changes in healthy adults: a scoping review. Neuropsychobiology.

[15] Sun Y, Hunt S, Sah P (2015). Norepinephrine and corticotropin-releasing hormone: partners in the neural circuits that underpin stress and anxiety. Neuron.

[16] Nikbakhtzadeh M, Ranjbar H, Moradbeygi K, Zahedi E, Bayat M, Soti M, Shabani M (2023). Cross-talk between the HPA axis and addiction-related regions in stressful situations. Heliyon.

[17] Zhang J, Li W, Braithwaite G, Blundell J (2024). Practice effects of a breathing technique on pilots' cognitive and stress associated heart rate variability during flight operations. Stress.

[18] Acuff S, MacKillop J, Murphy J (2023). A contextualized reinforcer pathology approach to addiction. Nat Rev Psychol.

[19] Kennedy DP, Osilla KC, Tucker JS (2022). Feasibility of a computer-assisted social network motivational interviewing intervention to reduce substance use and increase supportive connections among emerging adults transitioning from homelessness to housing. Addict Sci Clin Pract.

[20] Kwako LE, Bickel WK, Goldman D (2018). Addiction biomarkers: dimensional approaches to understanding addiction. Trends Mol Med.

[21] Narenji MA, Khazaee-Pool M, Iranpour A (2023). Development and psychometric properties of a health-promoting self-care behavior scale (HPSCB-S) in recovered patients from drug addiction. BMC Public Health.

[22] Watts AL, Latzman RD, Boness CL, Kotov R, Keyser-Marcus L, DeYoung CG, Krueger RF, Zald DH, Moeller FG, Ramey T (2023). New approaches to deep phenotyping in addictions. Psychol Addict Behav.

[23] World Health Organization, United Nations Office on Drugs and Crime United nations office on drugs and crime. International standards for the treatment of drug use disorders: revised edition incorporating results of field-testing. World Health Organization.

[24] Iasiello M, Muir-Cochrane E, van Agteren J, Fassnacht DB (2022). The effect of psychological distress on measurement invariance in measures of mental wellbeing. Int J Environ Res Public Health.

[25] Medina-Mora ME, Genis-Mendoza AD, Villatoro Velázquez JA, Bustos-Gamiño M, Bautista CF, Camarena B, Martínez-Magaña JJ, Nicolini H (2023). The prevalence of symptomatology and risk factors in mental health in Mexico: the 2016-17 ENCODAT cohort. Int J Environ Res Public Health.

[26] Valdez ES, Valdez L, Korchmaros J, Garcia DO, Stevens S, Sabo S, Carvajal S (2021). Socioenvironmental risk factors for adolescent marijuana use in a United States-Mexico border community. Am J Health Promot.

[27] Mendoza-Meléndez MÁ, Cepeda A, Frankeberger J, López-Macario M, Valdez A (2018). History of child sexual abuse among women consuming illicit substances in Mexico City. J Subst Use.

[28] National Institute on Drug Abuse, issuing body (2020). Common Comorbidities with Substance Use Disorders Research Report. National Institute on Drug Abuse.

[29] Motzkin JC, Baskin-Sommers A, Newman JP, Kiehl KA, Koenigs M (2014). Neural correlates of substance abuse: reduced functional connectivity between areas underlying reward and cognitive control. Hum Brain Mapp.

[30] Rykov Y, Thach T, Bojic I, Christopoulos G, Car J (2021). Digital biomarkers for depression screening with wearable devices: cross-sectional study with machine learning modeling. JMIR Mhealth Uhealth.

[31] Cheng Y, Petrides KV, Li J (2025). Estimating the minimum sample size for neural network model fitting-A monte carlo simulation study. Behav Sci (Basel).

[32] Gass JC, Tonkin S, Maguin E, Colder CR, Mahoney MC, Tiffany ST, Hawk LW (2024). A preliminary randomized trial of reinforcement contingencies to improve compliance with ecological momentary assessment. Transl Behav Med.

[33] Kathan A, Harrer M, Küster L, Triantafyllopoulos A, He X, Milling M, Gerczuk M, Yan T, Rajamani ST, Heber E, Grossmann I, Ebert DD, Schuller BW (2022). Personalised depression forecasting using mobile sensor data and ecological momentary assessment. Front Digit Health.

[34] Jacobson NC, Chung YJ (2020). Passive sensing of prediction of moment-to-moment depressed mood among undergraduates with clinical levels of depression sample using smartphones. Sensors (Basel).

[35] Buegler M, Harms R, Balasa M, Meier IB, Exarchos T, Rai L, Boyle R, Tort A, Kozori M, Lazarou E, Rampini M, Cavaliere C, Vlamos P, Tsolaki M, Babiloni C, Soricelli A, Frisoni G, Sanchez-Valle R, Whelan R, Merlo-Pich E, Tarnanas I (2020). Digital biomarker-based individualized prognosis for people at risk of dementia. Alzheimers Dement (Amst).

[36] Costa A, Milne R (2024). Detecting value(s): digital biomarkers for Alzheimer's disease and the valuation of new diagnostic technologies. Sociol Health Illn.

[37] Hajjar I, Okafor M, Choi JD, Moore E, Abrol A, Calhoun VD, Goldstein FC (2023). Development of digital voice biomarkers and associations with cognition, cerebrospinal biomarkers, and neural representation in early Alzheimer's disease. Alzheimers Dement (Amst).

[38] Lyall DM, Kormilitzin A, Lancaster C, Sousa J, Petermann-Rocha F, Buckley C, Harshfield EL, Iveson MH, Madan CR, McArdle R, Newby D, Orgeta V, Tang E, Tamburin S, Thakur LS, Lourida I, Llewellyn DJ, Ranson JM, Deep Dementia Phenotyping (DEMON) Network (2023). Artificial intelligence for dementia-applied models and digital health. Alzheimers Dement.

[39] Vanderlip C, Lee MD, Stark CEL (2024). Cognitive modeling of the mnemonic similarity task as a digital biomarker for alzheimer's disease. bioRxiv.

[40] Toader C, Dobrin N, Brehar F, Popa C, Covache-Busuioc R, Glavan LA, Costin HP, Bratu B, Corlatescu AD, Popa AA, Ciurea AV (2023). From recognition to remedy: the significance of biomarkers in neurodegenerative disease pathology. Int J Mol Sci.

[41] Choudhary S, Thomas N, Alshamrani S, Srinivasan G, Ellenberger J, Nawaz U, Cohen R (2022). A machine learning approach for continuous mining of nonidentifiable smartphone data to create a novel digital biomarker detecting generalized anxiety disorder: prospective cohort study. JMIR Med Inform.

[42] Cohen A, Naslund J, Lane E, Bhan A, Rozatkar A, Mehta UM, Vaidyam A, Byun AJS, Barnett I, Torous J (2025). Digital phenotyping data and anomaly detection methods to assess changes in mood and anxiety symptoms across a transdiagnostic clinical sample. Acta Psychiatr Scand.

[43] Jacobson NC, Bhattacharya S (2022). Digital biomarkers of anxiety disorder symptom changes: personalized deep learning models using smartphone sensors accurately predict anxiety symptoms from ecological momentary assessments. Behav Res Ther.

[44] Jacobson NC, Summers B, Wilhelm S (2020). Digital biomarkers of social anxiety severity: digital phenotyping using passive smartphone sensors. J Med Internet Res.

[45] Ryu J, Sükei E, Norbury A, H Liu S, Campaña-Montes JJ, Baca-Garcia E, Artés A, Perez-Rodriguez MM (2021). Shift in social media app usage during COVID-19 lockdown and clinical anxiety symptoms: machine learning-based ecological momentary assessment study. JMIR Ment Health.

[46] Fundoiano-Hershcovitz Y, Breuer Asher I, Ritholz MD, Feniger E, Manejwala O, Goldstein P (2023). Specifying the efficacy of digital therapeutic tools for depression and anxiety: retrospective, 2-cohort, real-world analysis. J Med Internet Res.

[47] Di Matteo D, Fotinos K, Lokuge S, Mason G, Sternat T, Katzman MA, Rose J (2021). Automated screening for social anxiety, generalized anxiety, and depression from objective smartphone-collected data: cross-sectional study. J Med Internet Res.

[48] Klein B, Nguyen H, McLaren S, Andrews B, Shandley K (2023). A fully automated self-help biopsychosocial transdiagnostic digital intervention to reduce anxiety and/or depression and improve emotional regulation and well-being: pre-follow-up single-arm feasibility trial. JMIR Form Res.

[49] Moshe I, Terhorst Y, Opoku Asare K, Sander LB, Ferreira D, Baumeister H, Mohr DC, Pulkki-Råback L (2021). Predicting symptoms of depression and anxiety using smartphone and wearable data. Front Psychiatry.

[50] Venkatesan A, Rahimi L, Kaur M, Mosunic C (2020). Digital cognitive behavior therapy intervention for depression and anxiety: retrospective study. JMIR Ment Health.

[51] Hu Y, Chen J, Chen J, Wang W, Zhao S, Hu X (2024). An ensemble classification model for depression based on wearable device sleep data. IEEE J Biomed Health Inform.

[52] Kishimoto T, Kinoshita S, Kikuchi T, Bun S, Kitazawa M, Horigome T, Tazawa Y, Takamiya A, Hirano J, Mimura M, Liang K, Koga N, Ochiai Y, Ito H, Miyamae Y, Tsujimoto Y, Sakuma K, Kida H, Miura G, Kawade Y, Goto A, Yoshino F (2022). Development of medical device software for the screening and assessment of depression severity using data collected from a wristband-type wearable device: SWIFT study protocol. Front Psychiatry.

[53] Pedrelli P, Fedor S, Ghandeharioun A, Howe E, Ionescu DF, Bhathena D, Fisher LB, Cusin C, Nyer M, Yeung A, Sangermano L, Mischoulon D, Alpert JE, Picard RW (2020). Monitoring changes in depression severity using wearable and mobile sensors. Front Psychiatry.

[54] Opoku Asare K, Terhorst Y, Vega J, Peltonen E, Lagerspetz E, Ferreira D (2021). Predicting depression from smartphone behavioral markers using machine learning methods, hyperparameter optimization, and feature importance analysis: exploratory study. JMIR Mhealth Uhealth.

[55] Ren B, Balkind EG, Pastro B, Israel ES, Pizzagalli DA, Rahimi-Eichi H, Baker JT, Webb CA (2023). Predicting states of elevated negative affect in adolescents from smartphone sensors: a novel personalized machine learning approach. Psychol Med.

[56] Sultana M, Al-Jefri M, Lee J (2020). Using machine learning and smartphone and smartwatch data to detect emotional states and transitions: exploratory study. JMIR Mhealth Uhealth.

[57] Ben-Zeev D, Chander A, Tauscher J, Buck B, Nepal S, Campbell A, Doron G (2021). A smartphone intervention for people with serious mental illness: fully remote randomized controlled trial of CORE. J Med Internet Res.

[58] Balliu B, Douglas C, Seok D, Shenhav L, Wu Y, Chatzopoulou D, Kaiser W, Chen V, Kim J, Deverasetty S, Arnaudova I, Gibbons R, Congdon E, Craske MG, Freimer N, Halperin E, Sankararaman S, Flint J (2024). Personalized mood prediction from patterns of behavior collected with smartphones. NPJ Digit Med.

[59] Choudhary S, Thomas N, Ellenberger J, Srinivasan G, Cohen R (2022). A machine learning approach for detecting digital behavioral patterns of depression using nonintrusive smartphone data (Complementary Path to Patient Health Questionnaire-9 Assessment): prospective observational study. JMIR Form Res.

[60] Hsu J, Wu C, Lin EC, Chen P (2024). MoodSensing: A smartphone app for digital phenotyping and assessment of bipolar disorder. Psychiatry Res.

[61] Jonathan GK, Abitante G, McBride A, Bernstein-Sandler M, Babington P, Dopke CA, Rossom RC, Mohr DC, Goulding EH (2024). LiveWell, a smartphone-based self-management intervention for bipolar disorder: intervention participation and usability analysis. J Affect Disord.

[62] Langholm C, Breitinger S, Gray L, Goes F, Walker A, Xiong A, Stopel C, Zandi P, Frye MA, Torous J (2023). Classifying and clustering mood disorder patients using smartphone data from a feasibility study. NPJ Digit Med.

[63] Bishop FM (2016). Relapse prediction: A meteorology-inspired mobile model. Health Psychol Open.

[64] Vairavan S, Rashidisabet H, Li QS, Ness S, Morrison RL, Soares CN, Uher R, Frey BN, Lam RW, Kennedy SH, Trivedi M, Drevets WC, Narayan VA (2023). Personalized relapse prediction in patients with major depressive disorder using digital biomarkers. Sci Rep.

[65] Holmgren JG, Morrow A, Coffee AK, Nahod PM, Santora SH, Schwartz B, Stiegmann RA, Zanetti CA (2022). Utilizing digital predictive biomarkers to identify veteran suicide risk. Front Digit Health.

[66] (2010). Comité consultivo nacional de normalización de innovación, desarrollo, tecnologías e información en salud. Diario Oficial de La Federación.

[67] General Data Protection Regulation. GDPR EU.

[68] (2024). ISO/IEC 29100:2024(en) Information technology — Security techniques — Privacy framework. International Organization for Standardization.

[69] Arias A, Ayuso M, Gil G, González I (1998). Cuestionario para Adultos de Experiencias Adversas en la Infancia.

[70] Beck AT, Robert AS, Brown GK (2011). Evaluación del Inventario de Depresión de Beck-II (BDI-II). Pearson Educación.

[71] Beck A, Steer R (2011). Inventario de Ansiedad de Beck. Consejo General de Colegios Oficiales de Psicólogos.

[72] Jiménez-Genchi A, Monteverde-Maldonado E, Nenclares-Portocarrero A (2008). Confiabilidad y análisis factorial de la versión en español del índice de calidad de sueño de Pittsburgh en pacientes psiquiátricos. Gaceta Médica Mexicana.

[73] Weschler David (2012). Escala de Inteligencia de Wechsler para Adultos-IV. Consejo General de la Psicología.

[74] Newcombe David Al, Humeniuk Rachel E, Ali R (2005). Validation of the World Health Organization Alcohol, Smoking and Substance Involvement Screening Test (ASSIST): report of results from the Australian site. Drug Alcohol Rev.

[75] Meule A, Nakovics H, Kübler A (2023). The mannheimer craving scale (MaCS): psychometric properties in a non-clinical sample and development of cut-off scores. OSF.

[76] Prochaska JO, DiClemente CC (1982). Motivación para el cambio de pacientes con trastorno por uso de sustancias URICA. Psiquiatria.com.

[77] Luo Wei, Phung Dinh, Tran Truyen, Gupta Sunil, Rana Santu, Karmakar Chandan, Shilton Alistair, Yearwood John, Dimitrova Nevenka, Ho Tu Bao, Venkatesh Svetha, Berk Michael (2016). Guidelines for developing and reporting machine learning predictive models in biomedical research: a multidisciplinary viewp. J Med Internet Res.

[78] Zhang D, Xu L, Liu X, Cui H, Wei Y, Zheng W, Hong Y, Qian Z, Hu Y, Tang Y, Li C, Liu Z, Chen T, Liu H, Zhang T, Wang J (2025). Eye movement characteristics for predicting a transition to psychosis: longitudinal changes and implications. Schizophr Bull.

[79] (2010). Ley federal de protección de datos personales en posesión de los particulares. Cámara de Diputados del H. Congreso de la Unión.

[80] (2011). Constitución política de los estados unidos Mexicanos - artículo 16. Diario oficial de La nación. Suprema Corte de Justicia de la Nación.

[81] Carretié L, Tapia M, López-Martín S, Albert J (2019). EmoMadrid: An emotional pictures database for affect research. Motiv Emot.

[82] (2024). Digital 2024: Mexico. DataReportal.

[83] Lee H, Cho C, Lee T, Jeong J, Yeom JW, Kim S, Jeon S, Seo JY, Moon E, Baek JH, Park DY, Kim SJ, Ha TH, Cha B, Kang H, Ahn Y, Lee Y, Lee J, Kim L (2023). Prediction of impending mood episode recurrence using real-time digital phenotypes in major depression and bipolar disorders in South Korea: a prospective nationwide cohort study. Psychol Med.

[84] Farhoumandi N, Mollaey S, Heysieattalab S, Zarean M, Eyvazpour R (2021). Facial emotion recognition predicts alexithymia using machine learning. Comput Intell Neurosci.

[85] Carreiro Stephanie, Ramanand Pravitha, Taylor Melissa, Leach Rebecca, Stapp Joshua, Sherestha Sloke, Smelson David, Indic Premananda (2024). Evaluation of a digital tool for detecting stress and craving in SUD recovery: An observational trial of accuracy and engagement. Drug Alcohol Depend.

[86] Padrós Blázquez F, Montoya Pérez KS, Bravo Calderón MA, Martínez Medina MP (2020). Propiedades psicométricas del Inventario de Ansiedad de Beck (BAI, Beck Anxiety Inventory) en población general de México. Ansiedad y Estrés.

[87] Rueda-Jaimes GE, Castro-Rueda VA, Rangel-Martínez-Villalba AM, Moreno-Quijano C, Martinez-Salazar GA, Camacho PA (2018). Validation of the beck hopelessness scale in patients with suicide risk. Rev Psiquiatr Salud Ment (Engl Ed).

[88] Roth E, Exeni S (2010). Exploración de la Validez y la Confiabilidad del Instrumento en la Medición de la Disposición al Cambio en Fumadores Habituales. Sistema de Información Científica Redalyc.

[89] Rosas R, Tenorio M, Pizarro M, Cumsille P, Bosch A, Arancibia S, Carmona-Halty M, Pérez-Salas C, Pino E, Vizcarra B, Zapata-Sepúlveda P (2014). Estandarización de la escala wechsler de inteligencia para adultos-cuarta edición en chile. Psykhe (Santiago).

[90] Guardia Serecigni J, Segura García L, Gonzalvo Cirac B, Trujols Albet J, Tejero Pociello A, Suárez González A, Martí Gil A (2004). [Validation study of the Multidimensional Alcohol Craving Scale (MACS)]. Med Clin (Barc).

[91] Casas Muñoz A, Velasco Rojano AE, Loredo Abdalá A (2022). Adaptación y validación de la prueba de detección de consumo de alcohol, tabaco y sustancias (ASSIST) en adolescentes mexicanos de una población semirrural. RIIAD.

[92] Ali FZ, Parsey RV, Lin S, Schwartz J, DeLorenzo C (2023). Circadian rhythm biomarker from wearable device data is related to concurrent antidepressant treatment response. NPJ Digit Med.

[93] Miskowiak K, Jespersen A, Obenhausen K, Hafiz P, Hestbæk E, Gulyas L, Kessing L, Bardram J (2021). Internet-based cognitive assessment tool: Sensitivity and validity of a new online cognition screening tool for patients with bipolar disorder. J Affect Disord.

[94] Lynham AJ, Jones IR, Walters JTR (2022). Web-based cognitive testing in psychiatric research: validation and usability study. J Med Internet Res.

[95] Ebner-Priemer UW, Mühlbauer E, Neubauer AB, Hill H, Beier F, Santangelo PS, Ritter P, Kleindienst N, Bauer M, Schmiedek F, Severus E (2020). Digital phenotyping: towards replicable findings with comprehensive assessments and integrative models in bipolar disorders. Int J Bipolar Disord.

[96] Bomyea JA, Parrish EM, Paolillo EW, Filip TF, Eyler LT, Depp CA, Moore RC (2021). Relationships between daily mood states and real-time cognitive performance in individuals with bipolar disorder and healthy comparators: a remote ambulatory assessment study. J Clin Exp Neuropsychol.

[97] Bai R, Xiao L, Guo Y, Zhu X, Li N, Wang Y, Chen Q, Feng L, Wang Y, Yu X, Xie H, Wang G (2021). Tracking and monitoring mood stability of patients with major depressive disorder by machine learning models using passive digital data: prospective naturalistic multicenter study. JMIR Mhealth Uhealth.

[98] Marwaa MN, Guidetti S, Ytterberg C, Kristensen HK (2024). Acceptability of two mobile applications to support cross-sectoral, person-centred and empowering stroke rehabilitation - a process evaluation. Ann Med.

[99] Yin Z, Yan J, Fang S, Wang D, Han D (2022). User acceptance of wearable intelligent medical devices through a modified unified theory of acceptance and use of technology. Ann Transl Med.

[100] Koychev I, Marinov E, Young S, Lazarova S, Grigorova D, Palejev D (2023). Identification of preclinical dementia according to ATN classification for stratified trial recruitment: A machine learning approach. PLoS One.

[101] Doukani A, Quartagno M, Sera F, Free C, Kakuma R, Riper H, Kleiboer A, Cerga-Pashoja A, van Schaik A, Botella C, Berger T, Chevreul K, Matynia M, Krieger T, Hazo J, Draisma S, Titzler I, Topooco N, Mathiasen K, Vernmark K, Urech A, Maj A, Andersson G, Berking M, Baños RM, Araya R (2024). Comparison of the working alliance in blended cognitive behavioral therapy and treatment as usual for depression in Europe: secondary data analysis of the E-COMPARED randomized controlled trial. J Med Internet Res.

[102] Comtois KA, Mata-Greve F, Johnson M, Pullmann MD, Mosser B, Arean P (2022). Effectiveness of mental health apps for distress during COVID-19 in US unemployed and essential workers: remote pragmatic randomized clinical trial. JMIR Mhealth Uhealth.

[103] Anýž J, Bakštein E, Dally A, Kolenič M, Hlinka J, Hartmannová T, Urbanová K, Correll CU, Novák D, Španiel F (2021). Validity of the aktibipo self-rating questionnaire for the digital self-assessment of mood and relapse detection in patients with bipolar disorder: instrument validation study. JMIR Ment Health.

